# Improvement of l-ornithine production by attenuation of *argF* in engineered *Corynebacterium glutamicum* S9114

**DOI:** 10.1186/s13568-018-0557-8

**Published:** 2018-02-24

**Authors:** Bin Zhang, Miao Yu, Ying Zhou, Bang-Ce Ye

**Affiliations:** 1 Laboratory of Biosystems and Microanalysis, State Key Laboratory of Bioreactor Engineering, East China University of Science and Technology, Shanghai, 200237 China; 2 School of Chemistry and Chemical Engineering, Shihezi University, Xinjiang, 832000 China

**Keywords:** *Corynebacterium glutamicum*, Terminator, Attenuation expression, l-Ornithine

## Abstract

l-Ornithine, a non-essential amino acid, has enormous industrial applications in food, pharmaceutical, and chemical industries. Currently, l-ornithine production is focused on microorganism fermentation using *Escherichia coli* or *Corynebacterium glutamicum*. In *C. glutamicum*, development of high l-ornithine producing *C. glutamicum* was achieved by deletion of *argF*, but was accompanied by growth deficiency and arginine auxotrophy. l-Arginine has been routinely added to solve this problem; however, this increases production cost and causes feedback inhibition of *N*-acetyl-l-glutamate kinase activity. To avoid the drawbacks of growth disturbance due to disruption of ArgF, strategies were adopted to attenuate its expression. Firstly, ribosome binding site substitution and start codon replacement were introduced to construct recombinant *C. glutamiucm* strains, which resulted in an undesirable l-ornithine production titer. Then, we inserted a terminator (*rrnB*) between *argD* and *argF*, which significantly improved l-ornithine production and relieved growth disturbance. Transcription analysis confirmed that a terminator can be used to downregulate expression of *argF* and simultaneously improve the transcriptional level of genes in front of *argF*. Using disparate terminators to attenuate expression of *argF*, an optimal strain (CO-9) with a T4 terminator produced 6.1 g/L of l-ornithine, which is 42.8% higher than that produced by strain CO-1, and is 11.2-fold higher than that of the parent CO strain. Insertion of terminators with gradient termination intensity can be a stable and powerful method to exert precise control of the expression level of *argF* in the development of l-ornithine producing strains, with potential applications in metabolic engineering and synthetic biology.

**Electronic supplementary material:**

The online version of this article (10.1186/s13568-018-0557-8) contains supplementary material, which is available to authorized users.

## Introduction

**Fig. 1:**
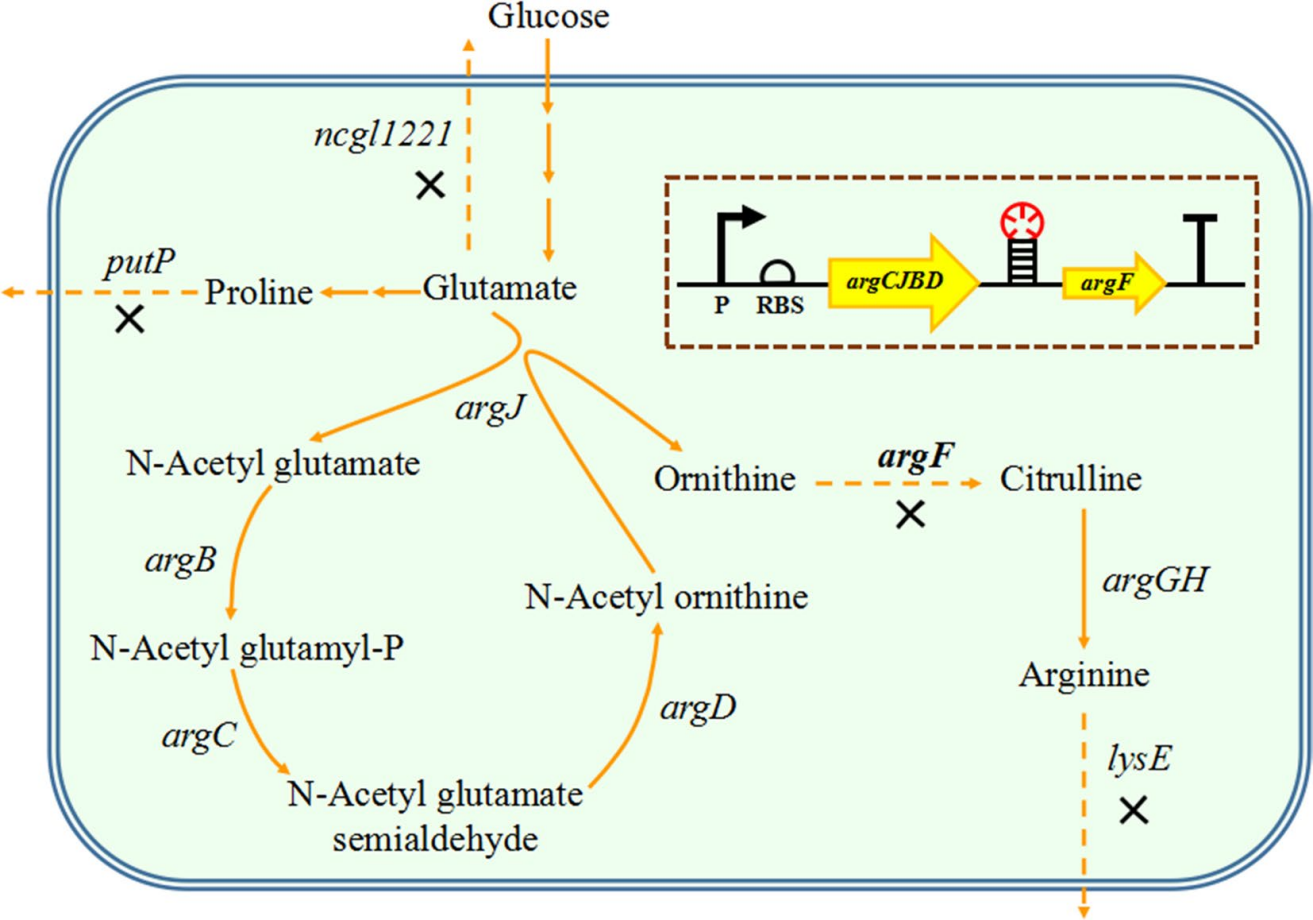
Metabolic pathways associated with ornithine biosynthesis in *C. glutamicum* and the metabolic engineering strategies of ornithine production. Yellow boxes represent the targeted modifications of the genes and the location of terminators. Products of the genes are: *argB* acetylglutamate kinase, *argC N*-acetyl-gamma-glutamylphosphate reductase, *argD* acetylornithine aminotransferase, *argF* ornithine carbamoyltransferase, *argG* arginine succinate synthase, *argH* arginosuccinase, *argJ* ornithine acetyltransferase/*N*-acetylglutamate synthase, *lysE* cncoding lysine/arginine transporter, *putP* encoding l-proline transporter, *ncgl1221* encoding glutamate transporter. Multiplication sign represents the deletion of relevant gene

l-Ornithine, a non-essential amino acid, plays an important role in urea cycle (Jiang et al. 2013); has various applications in the treatment of diseases such as liver diseases, gyrate atrophy, and cancers in humans; and is capable of improving athletic performance (Zajac et al. 2010). Due to its numerous applications, l-ornithine high-titer production has become an important task. Currently, due to the problems of high cost, complicated operation, and environmental harm, l-ornithine production by chemical means has generally been replaced by fermentation using *Escherichia coli* or *Corynebacterium glutamicum* (Hwang and Cho 2014; Jensen et al. 2015; Lee et al. 2010). l-Ornithine synthesis from l-glutamate consists of four enzymatic reactions, which involve the *argCJBDFR* operon in *C. glutamicum* (Fig. 1) (Kim et al. 2015). Among them, ornithine carbamoyl transferase (OTC), encoded by *argF*, is the key enzyme for converting l-ornithine to l-citrulline. Deletion of *argF* leads to l-ornithine accumulation and simultaneously, makes the strain auxotrophic for l-arginine (Lee and Cho 2006). Although addition of arginine into the medium can recover cell growth, it also leads to additional costs and feedback regulation. Thus, attenuation of expression of *argF* is a potential strategy for balancing l-ornithine production and cell growth.

To attenuate the expression of target genes, numerous strategies such as RBS modification, translational start codon exchange, promoter replacement, and RNA interference have been carried out and widely applied in the past decades (Man et al. 2016; Shen et al. 2017). RBS and changing of the translational start codon of the enzyme-coding genes directly affect the translation of the corresponding enzyme and have been applied in pathway engineering. Promoter replacement and RNA interference were useful strategies for regulation of the transcription process. Transcription is a process that includes recruitment of RNA polymerase (RNAP) to a promoter, synthesis of mRNA, and dissociation of RNAP at a terminator sequence. The terminator is an important component of the transcription process, which is known to be crucial for protein expression (Nakamura et al. 2015).

In this study, a *C. glutamicum* S9114 mutant strain, with deletion of *ncgl1221*, *lysE*, *putP*, and *argR*, was selected as the parent strain for the attenuation of *argF*. By analysis of l-ornithine production, cell growth, and the relative transcription level of the genes involved in the l-ornithine synthesis pathway, we confirmed that terminators can be used to downregulate *argF* expression and to improve l-ornithine production.

## Materials and methods

### Strains and growth conditions

The strains and plasmids used in this work are listed in Table 1. *E. coli* DH5α was used for DNA manipulation and plasmid construction. For recombinant DNA work, *E. coli* DH5α was cultivated at 37 °C in LB medium. If needed, kanamycin (50 mg/L for *E. coli* DH5α or 25 mg/L for *C. glutamicum* strains) was added to the medium. For l-ornithine production in *C. glutamicum*, a seed culture was prepared by inoculating cells into the seed medium [30 g glucose, 10 g yeast extract, 10 g corn steep liquor, 15 g (NH_4_)_2_SO_4_, 2.5 g MgSO_4_·7H_2_O, 1 g KH_2_PO_4_, 0.5 g K_2_HPO_4_, 0.5 g Na_2_HPO_4_, and 10 g CaCO_3_ per liter] and allowing them to grow for 11 h. Then, the seed culture was inoculated into 25 mL of the fermentation medium and the initial OD_600_ was adjusted to 1. Each liter of the fermentation medium consisted of 100 g glucose, 20 g corn steep liquor, 50 g (NH_4_)_2_SO_4_, 2.5 g MgSO_4_·7H_2_O, 1 g KH_2_PO_4_, 0.5 g K_2_HPO_4_, 0.5 g Na_2_HPO_4_, 0.02 g FeSO_4_·7H_2_O, 0.02 g MnSO_4_·4H_2_O, and 10 g CaCO_3_ (Zhang et al. 2017). For both media, the initial pH was adjusted to 7.0. All of the cultures were grown at 32 °C and 250 rpm on a rotary shaker, and samples were taken to monitor the l-ornithine production and biomass.

**Table 1:** Strains and plasmids used in this study

Strains and plasmids	Relevant characteristics	Sources or references
Strains		
*E. coli* DH5α	Clone host strain	Transgen
*C. glutamicum* S9114	Industrial strain for glutamate production	Mei et al. (2016)
CO	*C. glutamicum* S9114△*ncgl1221*△*lysE*△*putP*△*argR*	Our lab
CO-1	CO derivative with in-frame deletion of *argF*	This study
CO-2	CO derivative with replacement of RBS10 and A1G in *argF*	This study
CO-3	CO derivative with replacement of RBS50 and start A1G in *argF*	This study
CO-4	CO derivative with replacement of RBS100 A1G in *argF*	This study
CO-5	CO derivative with replacement of RBS500 and A1G in *argF*	This study
CO-6	CO derivative with insertion of terminator T1 in front of *argF*	This study
CO-7	CO derivative with insertion of terminator T2 in front of *argF*	This study
CO-8	CO derivative with insertion of terminator T3 in front of *argF*	This study
CO-9	CO derivative with insertion of terminator T4 in front of *argF*	This study
CO-10	CO derivative with insertion of terminator T5 in front of *argF*	This study
CO-11	CO derivative with insertion of terminator T6 in front of *argF*	This study
CO-12	CO derivative with insertion of terminator T7 in front of *argF*	This study
CO-13	CO derivative with insertion of terminator T8 in front of *argF*	This study
CO-14	CO derivative with insertion of terminator T9 in front of *argF*	This study
CO-15	CO derivative with insertion of terminator T10 in front of *argF*	This study
CO-16	CO derivative with insertion of terminator T11 in front of *argF*	This study
CO-17	CO derivative with insertion of terminator T12 in front of *argF*	This study
CO-18	CO derivative with insertion of terminator T13 in front of *argF*	This study
CO-19	CO derivative with insertion of terminator T14 in front of *argF*	This study
CO-20	CO derivative with insertion of terminator T15 in front of *argF*	This study
CO-21	CO derivative with insertion of terminator T16 in front of *argF*	This study
CO-22	CO derivative with insertion of terminator T17 in front of *argF*	This study
CO-23	CO derivative with insertion of terminator T18 in front of *argF*	This study
Plasmids		
pK18*mobsacB*	Mobilizable vector, allows for selection of double crossover in *C. glutamicum,* Km^R^, *sacB*	Zhang et al. (2015)
pXMJ19	A shuttle expression vector, Cm^R^	Jakoby et al. (1999)
pK18-△*argF*	A derivative of pK18*mobsacB*, harboring △*argF* fragment	This study
pK18-*argF*10-G	A derivative of pK18*mobsacB*, harboring the fragment of *argF* (10 au) RBS change and start codon replacement with GTG	This study
pK18-*argF*50-G	A derivative of pK18*mobsacB*, harboring the fragment of *argF* (50 au) RBS change and start codon replacement with GTG	This study
pK18-*argF*100-G	A derivative of pK18*mobsacB*, harboring the fragment of *argF* (100 au) RBS change and start codon replacement with GTG	This study
pK18-*argF*500-G	A derivative of pK18*mobsacB*, harboring the fragment of *argF* (500 au) RBS change and start codon replacement with GTG	This study
pK18-T1	A derivative of pK18*mobsacB*, harboring the fragment of inserting T1 terminator in front of *argF*	This study
pK18-T2	A derivative of pK18*mobsacB*, harboring the fragment of inserting T2 terminator in front of *argF*	This study
pK18-T3	A derivative of pK18*mobsacB*, harboring the fragment of inserting T3 terminator in front of *argF*	This study
pK18-T4	A derivative of pK18*mobsacB*, harboring the fragment of inserting T4 terminator in front of *argF*	This study
pK18-T5	A derivative of pK18*mobsacB*, harboring the fragment of inserting T5 terminator in front of *argF*	This study
pK18-T6	A derivative of pK18*mobsacB*, harboring the fragment of inserting T6 terminator in front of *argF*	This study
pK18-T7	A derivative of pK18*mobsacB*, harboring the fragment of inserting T7 terminator in front of *argF*	This study
pK18-T8	A derivative of pK18*mobsacB*, harboring the fragment of inserting T8 terminator in front of *argF*	This study
pK18-T9	A derivative of pK18*mobsacB*, harboring the fragment of inserting T9 terminator in front of *argF*	This study
pK18-T10	A derivative of pK18*mobsacB*, harboring the fragment of inserting T10 terminator in front of *argF*	This study
pK18-T11	A derivative of pK18*mobsacB*, harboring the fragment of inserting T11 terminator in front of *argF*	This study
pK18-T12	A derivative of pK18*mobsacB*, harboring the fragment of inserting T12 terminator in front of *argF*	This study
pK18-T13	A derivative of pK18*mobsacB*, harboring the fragment of inserting T13 terminator in front of *argF*	This study
pK18-T14	A derivative of pK18*mobsacB*, harboring the fragment of inserting T14 terminator in front of *argF*	This study
pK18-T15	A derivative of pK18*mobsacB*, harboring the fragment of inserting T15 terminator in front of *argF*	This study
pK18-T16	A derivative of pK18*mobsacB*, harboring the fragment of inserting T16 terminator in front of *argF*	This study
pK18-T17	A derivative of pK18*mobsacB*, harboring the fragment of inserting T17 terminator in front of *argF*	This study
pK18-T18	A derivative of pK18*mobsacB*, harboring the fragment of inserting T18 terminator in front of *argF*	This study

Superscript ‘‘R’’ indicates resistance to the following antibiotics: *Km* kanamycin, *Cm* chloramphenicol

### Construction of recombinant plasmids and strains

All recombinant strains were derived from *C. glutamicum* S9114 (Mei et al. 2016), which also stored at Shanghai Industrial Institute of Microorganisms (SIIM), Shanghai, China with the storage number as SIIM B460 and China Center of Industrial Culture Collection (CICC) with the registration number of CICC 20935. The homologous *sacB* recombination system was used to introduce modulations into the chromosome as described previously (Kim et al. 2015; Niebisch and Bott 2001). To disrupt *argF* in *C. glutamicum*, the upstream region and downstream region of *argF* were PCR amplified and cloned into the *Hin*dIII/*Xba*I sites of pK18*mobsacB* by Gibson assembly to generate the recombinant plasmid pK18-△*argF*. In addition, for RBS replacement in the chromosome, the recombinant plasmids with upstream and downstream fragments, an artificial, synthetic RBS, and A1G replacement were also PCR amplified and cloned into pK18*mobsacB*. Synthetic RBSs with different translation initiation strengths were designed by an RBS Calculator (Tian and Salis 2015) (https://www.denovodna.com/software/doLogin) and inserted among the homologous arms by overlap PCR. The sequences of the synthetic RBSs are listed in Additional file 1: Table S2. Moreover, for insertion of the terminator in front of *argF*, terminators derived from a previous study (Chen et al. 2013) were added between the upstream and downstream sequences by overlap PCR and then cloned into plasmid pK18*mobsacB*. All of the recombinant plasmids were transformed into *C. glutamicum* cells by electroporation. After two rounds of homologous recombination, engineered *C. glutamicum* with the corresponding chromosomal modifications were verified by PCR. All of the primers used in this study are listed in Additional file 1: Table S1.

### Quantitative real-time (RT) PCR

RT-PCR assays were conducted as described in our previous study (Liao et al. 2015). Total RNA from *C. glutamicum* cells was extracted during the exponential phase using an RNA extraction kit (Tiangen Biotech Co., Ltd., Beijing, China), and the RNA concentration was determined by a microplate reader (BioTek Instruments, Winooski, VT, USA). The cDNA was synthesized using the PrimeScript RT Reagent Kit with gDNA Eraser (TaKaRa, Shiga, Japan) and a Touch Real-Time PCR System (Bio-Rad Hercules, CA, USA), using the SYBR Premix Ex TaqTM II (TaKaRa, Shiga, Japan) on a Bio-Rad CFX96. cDNA (100 ng) was used as template. The PCR conditions were: 94 °C for 30 min, then 45 cycles at 94 °C for 5 s and 60 °C for 30 min. The data were normalized as per the 16S rRNA expression. The primers for RT-PCR are presented in the Additional file 1.

### Analytical procedures

Cell growth was monitored by measuring the optical density of the culture at 600 nm (OD_600_) using an spectrophotometer, after dilution of the culture with 0.125 mol/L HCl to dissolve CaCO_3_ (Hao et al. 2016). The production of l-ornithine was determined by ninhydrin colorimetry, as described previously (Chinard 1952).

## Results

### l-Ornithine accumulation and cell growth deficiency led by deletion of *argF*

**Fig. 2:**
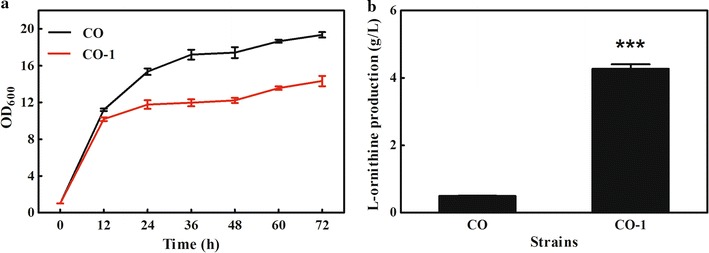
Cell growth and l-ornithine production of the strains CO and CO-1. **a** Growth curves under 72 h in shake flasks. **b** l-Ornithine production at 48 h of incubation. The error bars represent the standard deviation of samples. ***p ≤ 0.001

**Fig. 3:**
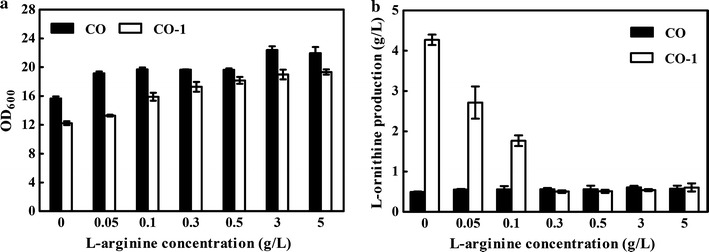
Fermentation of the strains CO and CO-1 with addition of disparate l-arginine concentration into the medium. **a** Cell growth. **b** l-Ornithine production at 48 h in shake flasks

*ArgF*, encoding ornithine carbamoyl transferase (OTC), plays a critical role in the biodegradation of l-ornithine. To accumulate l-ornithine, *argF* was deleted in strain CO, resulting in strain CO-1. Then, strain CO-1 was cultivated in a shaking flask, and the fermentation data revealed that 4.27 g/L of l-ornithine was detected in the broth after 48 h of incubation. However, the cell growth of CO-1 was 22% lower than that of the parent CO strain (Fig. 2). Recovery of cell growth by the addition of l-arginine into the broth has been frequently used as a strategy to relieve the growth disturbance caused by *argF* deletion. Therefore, to relieve the growth deficiency, an l-arginine addition experiment was performed. As shown in Fig. 3a, growth of strain CO-1 gradually recovered with the supplementation of l-arginine, which indicated that the growth disturbance was caused by deficiency of l-arginine. However, no significant changes in l-ornithine production were observed after addition of l-arginine in the CO strain, while the l-ornithine production titer of strain CO-1 was reduced. As shown in Fig. 3b, when l-arginine was added up to a concentration of 5 g/L, the l-ornithine production of strain CO-1 dropped from 4.27 to 0.61 g/L. These results suggested that deletion of *argF* in the engineered strain CO-1 led to growth deficiency, which was relieved by l-arginine supplementation, but addition of l-arginine into the fermentation broth inhibited l-ornithine production in this strain.

### RBS optimization was undesirable for attenuating the expression of *argF* to promote l-ornithine production

**Fig. 4:**
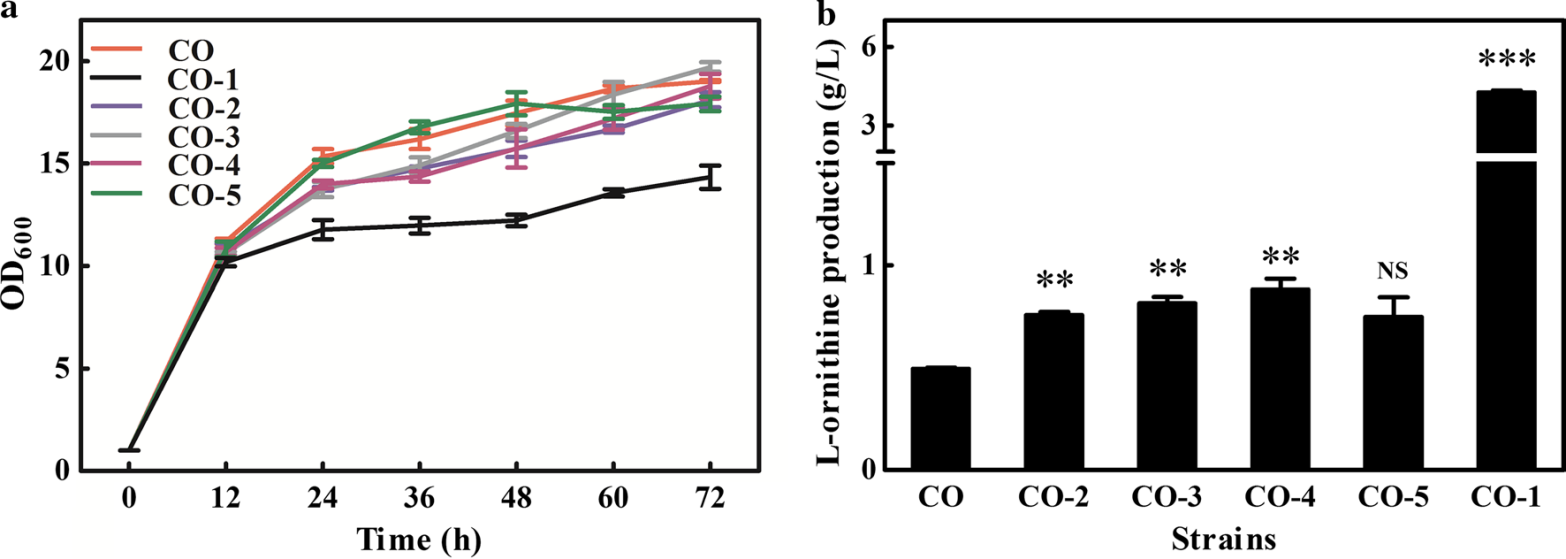
Cell growth and l-ornithine production of the strains with changing RBS site and replacing start codon of argF. **a** Growth curves under 72 h of Shake flask culture. **b** l-Ornithine production at 48 h of incubation. The error bars represent the standard deviation of samples. Compared with the controlling strain CO. NS means not significant. **p ≤ 0.05 and ***p ≤ 0.001

Based on the results of the l-arginine addition experiments and after taking the cost into consideration, we found that supplementation of the medium with l-arginine was unsuitable for relieving the growth disturbance brought by arginine auxotroph. Then, inspired by a previous study that fine-tuned ornithine transcarbamoylase activity using a plasmid addiction system to improve putrescine production (Schneider et al. 2012), we intended to downregulate *argF* expression and explore an appropriate expression model that balanced the cell growth and l-ornithine production. Thus, RBS substitution and start codon replacement were carried out to attenuate the expression of *argF*. The predicted natural RBS strength of *argF* is 35364.4 au. Therefore, we replaced the natural RBS with weaker RBSs with different initial translation strengths (10, 50, 100, and 500 au), and the start codon ATG was replaced with GTG, generating strains CO-2, CO-3, CO-4, and CO-5. As shown in Fig. 4, l-ornithine production by these strains showed obvious improvement, compared to the CO strain, but the highest titer produced by strain CO-4 was only 1 g/L, which was three-fold lower than that produced by CO-1 (4.27 g/L). It was concluded that attenuating the expression of *argF* through RBS optimization and start codon replacement was undesirable for l-ornithine accumulation.

### Improvement of l-ornithine production by insertion of a terminator in front of *argF*

**Fig. 5:**
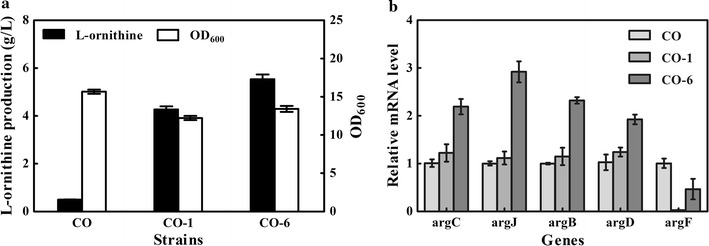
Fermentation data and relative transcription levels of selected genes of the strains CO, CO-1 and CO-6. **a** Cell growth and l-ornithine production of the strains. **b** Comparison of the transcriptional levels of l-ornithine biosynthesis genes

ArgR, a negative regulatory protein of the *argCJBDFR* operon, was disrupted previously, which led to a higher transcription level of *argCJBDF* in CO. The increased transcription level of *argF* might explain why replacement of the RBS and start codon could not lead to the desired l-ornithine production level. Therefore, we continued to insert a terminator (*rrnB* from plasmid pXMJ19) before *argF* to reduce the transcription level of *argF*, generating strain CO-6. Interestingly, after 48 h of shake flask fermentation, strain CO-6 produced 5.53 g/L of l-ornithine, which was 29.5% higher than the production level of the *argF* deletion strain CO-1 (4.27 g/L) (Fig. 5a). Cell growth also increased from an OD_600_ of 12.23 to an OD_600_ of 13.42. To investigate the mechanism behind this phenomenon, the transcription levels of the genes involved in l-ornithine synthesis were analyzed. The expression level of *argF* in strain CO-1 dropped to zero. Compared with the parent strain CO, the relative transcriptional level of *argF* in strain CO-6 was reduced to 47%. For the terminator upstream of the genes, the expression levels of *argC*, *argJ*, *argB*, and *argD* in strain CO-6 increased 2.17-, 2.91-, 2.32-, and 1.88-fold, respectively, while no corresponding changes were detected in strain CO-1 (Fig. 5b). These results suggested that increased expression of the *argCJBD* operon stimulated l-ornithine production, and the leaky expression of *argF* contributed to the improvement of cell growth.

### Improvement of l-ornithine production by optimizing the terminators

**Fig. 6:**
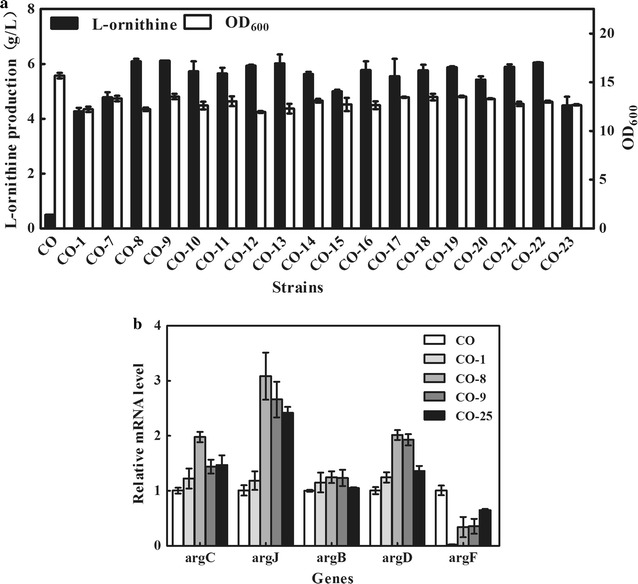
Fermentation data and relative transcription levels of selected genes in the strains with terminators at 48 h in shake flasks. **a** Cell growth and l-ornithine production of the strains. **b** Comparison of the transcriptional levels of l-ornithine biosynthesis genes

In order to increase l-ornithine production with an improvement in cell growth, seventeen terminators discovered by Chen et al. (Chen et al. 2013) (Additional file 1: Table S3) with different termination strengths were selected and inserted into the chromosome of strain CO, resulting in 17 mutant strains (CO-7 to CO-23). Fermentation experiments were carried out to evaluate the effect of these modifications on l-ornithine production and cell growth. As shown in Fig. 6a, the yield of l-ornithine, produced by strain CO-9, was 6.1 g/L, which was 42.8% higher than that by strain CO-1 (4.27 g/L). Compared with CO-1, the cell growth of CO-9 also improved from OD_600_ = 12.2 to OD_600_ = 13.5, after 48 h of incubation. The *argF* expression levels of the recombinant strains CO-8, CO-9, and CO-25, with terminator strengths of 239.91, 216.60, and 10.94, were 34, 35, and 65%, respectively, indicating that the expression level of *argF* decreased with improved terminator strength. In addition, insertion of a terminator in front of *argF* was an efficient strategy for improving l-ornithine production, by slightly relieving the growth disturbance, by blocking l-ornithine degradation. Moreover, these results also suggested that addition of a terminator could act as a reliable method for controlling gene expression and are a potential genetic engineering tool for *C. glutamicum*.

## Discussion

Based on our current knowledge, gene knockout is an effective strategy in genetic engineering for developing high target-compound producing strains. However, deletion of growth-coupled genes can induce some undesired results, such as a growth deficiency or extra nutritional requirements, which are undesirable for industrial fermentation. The same phenomenon of biomass deficiency caused by deletion, *argF* specifically here, was observed in the study on l-arginine auxotroph in engineered *C. glutamicum* S9114, which employed a common strategy used in the construction of engineered strains for l-ornithine production (Hwang et al. 2008; Zhang et al. 2017). However, addition of l-arginine caused feedback inhibition of *N*-acetyl-l-glutamate kinase (NAGK) enzyme activity, which reduced the yield of l-ornithine, consistent with the results observed in a previous study (Kim et al. 2015). This problem was solved by overexpression of an anti-feedback inhibited NAGK from *C. glutamicum* ATCC 21831; however, the cost of l-arginine addition and the genetic instability from using a plasmid hampered its industrial application in l-ornithine production. Thus, to address these problems, attenuation of the expression of *argF*, instead of direct gene deletion, was explored to balance l-ornithine production and cell growth. First, we chose an RBS optimization strategy, which is a promising metabolic engineering method that has been applied to construct a pathway for various compounds in previous reports (Sun et al. 2016; Veetil et al. 2017), to attenuate the expression of *argF*. However, replacement of the original RBS of *argF* with sequences with low translation initiation intensity could not achieve the desired l-ornithine production, though cell growth was unaffected.

We speculate that deletion of ArgR inactivated the feedback inhibition of the transcription of the *argCJBDFR* operon in the l-arginine biosynthetic pathway (Chen et al. 2014; Lee et al. 2011; Xu et al. 2013; Yim et al. 2011), which covered the attenuation effect of RBS optimization and A1G replacement and was unable to tightly control the metabolic flow of l-ornithine degradation. The transcription of *argF* plays a more important role in l-ornithine catabolism. In view of this, to reduce the transcription level of *argF*, transcription terminators, which are known to play critical roles in regulating natural genetic systems and implementing synthetic genetic logic, are employed (Cambray et al. 2013). Interestingly, compared with the control strain with *argF* deletion, l-ornithine production titer was improved by 29.5% after insertion of the *rrnB* terminator in front of *argF*. According to previous study, the terminators can not only stop the transcription process, but also function to prolong the mRNA half-life period, thus, stimulating the expression of upstream genes (Curran et al. 2013; Uzelac et al. 2015). Combined with analysis of transcriptional levels, we confirmed that inserting a terminator in front of *argF* causes high expression of the *argCJBD* gene cluster. Overexpression of the *argCJBD* operon is essential for l-ornithine biosynthesis (Hwang et al. 2008). In addition, independent overexpression of ArgJ in *C. glutamicum* 1006Δ*argR* was reported to significantly improve l-ornithine fermentation (Hao et al. 2016). Therefore, the improvement of l-ornithine production was attributed to the activated expression of the upstream genes by the terminator. A similar conclusion had been drawn in a previous work, where integration of the transcription terminator downstream of the target genes caused a significant improvement of the expression level in a plasmid (Ito et al. 2015).

In conclusion, numerous efforts have been made to attenuate the expression of *argF*, at both the translation and transcription levels, with the aim to increase the production of l-ornithine. Those strategies with several advantages over the previous *argF* deletion can be employed to the existing strains to further improvement of l-ornithine production and may be widely applicable to fine turning the expression of other growth coupled enzymes. To our knowledge, this is the first study in which a terminator-based strategy has been successfully developed to reduce the expression of *argF* and enhance l-ornithine production. The relative mRNA analysis provides valuable information for enhancing the expression of the upstream genes by insertion of terminator, which may find use in the construction of other middle metabolite producing strains. However, we failed to determine the optimal expression of *argF* for l-ornithine production without cell growth disturbance, and the l-ornithine production titer achieved in this study was relatively lower than that reported by other studies (Kim et al. 2015). However, insertion of a developed terminator, as performed in this study, is a novel strategy for improving l-ornithine production, which has potential application in engineering other high l-ornithine strains. The successful application of a terminator to regulate upstream and downstream gene expression also hinted at the possible applications of terminators in metabolic engineering to produce valuable products, which would enrich the metabolic engineering strategies for selective strain development. In our recent work, this strategy was employed for development of even higher l-ornithine producing strains by attenuation of *proB* and *ncgl2228* Zhang et al. (2017). According to our experience, insertion of terminator is a convenient and easy method than gene deletion in the development of engineered *C. glutamicum* strains. We expect this technology to be extended to allow more laboratories to use it. In our next work, more experiments and examples based on terminators will be provided in the development of high l-ornithine producing strains.

## Additional file

**Additional file 1.** Primers used in this study.

## Abbreviations

RBS: ribosome bind site
OTC: ornithine carbamoyl transferase
RNAP: RNA polymerase
NAGK: *N*-acetyl-l-glutamate kinase
