# Supplementary material for: Improvement of l-ornithine production by attenuation of argF in engineered Corynebacterium glutamicum S9114
Source: AMB Express. 2018 Feb 24;8:26. doi: 10.1186/s13568-018-0557-8 (PMC6890880; doi:10.1186/s13568-018-0557-8)
Supplement: Supplementary file 1 — Additional file 1. Primers used in this study. [file 13568_2018_557_MOESM1_ESM.docx]

**Additional file 1**

**AMB express**

**Improvement of L-ornithine production by attenuation of *argF* in engineered *Corynebacterium glutamicum* S9114**

Bin Zhang^a1^, Miao Yu^a1^, Ying Zhou^a*^, Bang-Ce Ye^ab*^

^a^Laboratory of Biosystems and Microanalysis, State Key Laboratory of Bioreactor Engineering, East China University of Science and Technology, Shanghai, 200237, China

^b^School of Chemistry and Chemical Engineering, Shihezi University, Xinjiang 832000, China

^1^ These two authors contributed equally to this work.

^*^Address correspondence to Ying Zhou, [zhouying@ecust.edu.cn](mailto:zhouying@ecust.edu.cn) and Bang-Ce Ye, [bcye@ecust.edu.cn](mailto:bcye@ecust.edu.cn)

Meilong Road 130, Shanghai, 200237, China. Tel/Fax: 0086-21-6425-2094

TABLE S1 Primers and their sequences in this study

| **Primers** | **Sequence (5’-3’)** |
| --- | --- |
| *argF*-up-F | CGGTACCCGGGGATCCTCTAGCAAACGAGGCTGCTTTCAAGAT |
| argF-up-R (10G) | ATTCTCGGTTGAGCGGAATAGCTTTATGCGATTGTCTCGGCAAT |
| *argF*-down-F (10G) | CTATTCCGCTCAACCGAGAATAGCGCCTAAGGGTGTGACTTCACAACCACAGGTTCG |
| *argF*-down-R | AACGACGGCCAGTGCCAAGCTTTGGACGCTGGTCCATCAATCAC |
| *argF*-10-F | ATTCCGCTCAACCGAGAATAG |
| *argF*-up-R (50G) | GACCTAAGGTTATAGGAGTAGATAGTCTTTATGCGATTGTCTCGGCAAT |
| *argF*-down-F (50G) | ACTATCTACTCCTATAACCTTAGGTCGTGACTTCACAACCACAGGTTCG |
| *argF*-50-F | ACTATCTACTCCTATAACCTTAGGTC |
| *argF*-up-R (100G) | GAGCGGTTTTCCTTATGCGCCTACTTTATGCGATTGTCTCGGCAAT |
| *argF*-down-F (100G) | TAGGCGCATAAGGAAAACCGCTCTTTCCGCTTAGTGACTTCACAACCACAGGTTCG |
| *argF*-100-F | CGCATAAGGAAAACCGCTCTTTC |
| *argF*-up-R (500G) | AGGAGTAGTGGAAAGTGTAATTACTACTTTATGCGATTGTCTCGGCAAT |
| *argF*-down-F (500G) | TAGTAATTACACTTTCCACTACTCCTACTAGTGACTTCACAACCACAGGTTCG |
| *argF*-500-F | GTAATTACACTTTCCACTACTCCTAC |
| *argF*-up-R (knockout) | GTCGTCGACGCGTCTTTATGCGATTGTCTCGGCAAT |
| *argF*-down-F (knockout) | TCGCATAAAGACGCGTCGACGACTAAGACATGTCCCTTGGCTCAAC |
| *argF*-down-R (knockout) | AACGACGGCCAGTGCCAAGCTCTTGATGGTTGGCAGGCAGTAC |
| *argF*-up-R (CO6) | CTTTATGCGATTGTCTCGGCAAT |
| T1-F(CO6) | TTGCCGAGACAATCGCATAAAGGCTCGAATTCAGCTTGGCTGTT |
| T1-R(CO6) | ATTCTCGGTTGAGCGGAATAGAGAGTTTGTAGAAACGCAAAAAGGC |
| *argF*-up-R(CO7) | GTGCGGGCTTTTTTCTGTGTTTCCCTTTATGCGATTGTCTCGGCAAT |
| *argF*-down-F2(CO7) | GGAAACACAGAAAAAAGCCCGCACCTGACAGTGCGGGCTTTTTTTTTCG |
| *argF*-down-F1(CO7) | GACAGTGCGGGCTTTTTTTTTCGACCAAAGGCTATTCCGCTCAACCGAGAA |
| *argF*-up-R(CO8) | CTTTTCTGGAATTTGGTACCGAGCTTTATGCGATTGTCTCGGCAAT |
| *argF*-down-F2(CO8) | CTCGGTACCAAATTCCAGAAAAGAGGCCTCCCGAAAGGGGGGCCTTTTTTC |
| *argF*-down-F1(CO8) | TCCCGAAAGGGGGGCCTTTTTTCGTTTTGGTCCCTATTCCGCTCAACCGAGAA |
| *argF*-up-R(CO9) | TTTTGGTACCGAGCTTTATGCGATTGTCTCGGCAAT |
| *argF*-down-F2(CO9) | ATCGCATAAAGCTCGGTACCAAAAAAAAAAAAAAAGACGCTGAAAAGCGTCTTTTTTCG |
| *argF*-down-F1(CO9) | GCTGAAAAGCGTCTTTTTTCGTTTTGGTCCCTATTCCGCTCAACCGAGAA |
| *argF*-up-R(CO10) | CACCCGTTAGGGTGTTTTTTTTTTTTCTTTATGCGATTGTCTCGGCAAT |
| *argF*-down-F2(CO10) | AAAAAAAAAAAACACCCTAACGGGTGTTTTTTTTTTTTTGGTCT |
| *argF*-down-F1(CO10) | CGGGTGTTTTTTTTTTTTTGGTCTCCCCTATTCCGCTCAACCGAGAA |
| *argF*-up-R(CO11) | CTCTTTTCTGGAATTTGGTACCGAGCTTTATGCGATTGTCTCGGCAAT |
| *argF*-down-F2(CO11) | CTCGGTACCAAATTCCAGAAAAGAGGCCGCGAAAGCGGCCTTTTTTCGT |
| *argF*-down-F1(CO11) | GCGAAAGCGGCCTTTTTTCGTTTTGGTCCCTATTCCGCTCAACCGAGAA |
| *argF*-up-R(CO12) | GATTTGGGAGGCCTTATTGTTCGTCCTTTATGCGATTGTCTCGGCAAT |
| *argF*-down-F2(CO12) | GACGAACAATAAGGCCTCCCAAATCGGGGGGCCTTTTTTATTG |
| *argF*-down-F1(CO12) | AAATCGGGGGGCCTTTTTTATTGATAACAAAACTATTCCGCTCAACCGAGAA |
| *argF*-up-R(CO13) | CTTTTCTGGAATTTGGTACCGAGCTTTATGCGATTGTCTCGGCAAT |
| *argF*-down-F2(CO13) | CTCGGTACCAAATTCCAGAAAAGAGACGCTGAAAAGCGTCTTT |
| *argF*-down-F1(CO13) | AAGAGACGCTGAAAAGCGTCTTTTTTCGTTTTGGTCCCTATTCCGCTCAACCGAGAA |
| *argF*-up-R(CO14) | CTCTTTTCTGGAATTTGGTACCGAGCTTTATGCGATTGTCTCGGCAAT |
| *argF*-down-F2(CO14) | CTCGGTACCAAATTCCAGAAAAGAGACGCTTTTAGAGCGTCTTT |
| *argF*-down-F1(CO14) | AGACGCTTTTAGAGCGTCTTTTTTCGTTTTGGTCCCTATTCCGCTCAACCGAGAA |
| *argF*-up-R(CO15) | GTTAGGGAGGCCTTATTGTTCGTCCTTTATGCGATTGTCTCGGCAAT |
| *argF*-down-F2(CO15) | GACGAACAATAAGGCCTCCCTAACGGGGGGCCTTTTTTATTG |
| *argF*-down-F1(CO15) | CCTAACGGGGGGCCTTTTTTATTGATAACAAAACTATTCCGCTCAACCGAGAA |
| *argF*-up-R(CO16) | CTTCCGGGGGCTTTCTCATGCGTTCTTTATGCGATTGTCTCGGCAAT |
| *argF*-down-F2(CO16) | AACGCATGAGAAAGCCCCCGGAAGATCACCTTCCGGGGGCTTTTTT |
| *argF*-down-F1(CO16) | GATCACCTTCCGGGGGCTTTTTTATTGCGCCTATTCCGCTCAACCGAGAA |
| *argF*-up-R(CO17) | GTCTTTTCTGGAATTTGGTACCGAGCTTTATGCGATTGTCTCGGCAAT |
| *argF*-down-F2(CO17) | CTCGGTACCAAATTCCAGAAAAGACACCCGAAAGGGTGTTTTTTCG |
| *argF*-down-F1(CO17) | ACACCCGAAAGGGTGTTTTTTCGTTTTGGTCCCTATTCCGCTCAACCGAGAA |
| *argF*-up-R(CO18) | ACCCGTTAGGGTGTTCAATAATTGGCTTTATGCGATTGTCTCGGCAAT |
| *argF*-down-F2(CO18) | CCAATTATTGAACACCCTAACGGGTGTTTTTTTGT |
| *argF*-down-F1(CO18) | CACCCTAACGGGTGTTTTTTTGTTTCTGGTCTCCCCTATTCCGCTCAACCGAGAA |
| *argF*-up-R(CO19) | CGATTTGGGTGTTCAATAATTGGCTTTATGCGATTGTCTCGGCAAT |
| *argF*-down-F2(CO19) | CCAATTATTGAACACCCAAATCGGGTGTTTTTTTG |
| *argF*-down-F1(CO19) | CACCCAAATCGGGTGTTTTTTTGTTTCTGGTCTCCCCTATTCCGCTCAACCGAGAA |
| *argF*-up-R(CO20) | CGTTAGCCGCTTCAATAATTGGCTTTATGCGATTGTCTCGGCAAT |
| *argF*-down-F2(CO20) | CCAATTATTGAAGCGGCTAACGCCGCTTTTTTTGTTT |
| *argF*-down-F1(CO20) | GGCTAACGCCGCTTTTTTTGTTTCTGGTCTCCCCTATTCCGCTCAACCGAGAA |
| argF-up-R(CO21) | GGACCTTTCGGTGCGGGGGTCTTCTTTATGCGATTGTCTCGGCAAT |
| argF-down-F(CO21) | AAGACCCCCGCACCGAAAGGTCCGGGGGTTTTTTTTCTATTCCGCTCAACCGAGAA |
| argF-up-R(CO22) | AAAAGGGCGATCATCTGACCGCCCCTTTATGCGATTGTCTCGGCAAT |
| argF-down-F(CO22) | GCGGTCAGATGATCGCCCTTTTTTTTTCTATTCCGCTCAACCGAGAA |
| *argF*-up-R(CO23) | ACGCCGGTATTTTTATTGGCTCGTCTTTATGCGATTGTCTCGGCAAT |
| *argF*-down-F2(CO23) | ACGAGCCAATAAAAATACCGGCGTTATGCCGGTATTTTTTTACG |
| *argF*-down-F1(CO23) | CGTTATGCCGGTATTTTTTTACGAAAGACTATTCCGCTCAACCGAGAA |
| *argB*-F | CAACTCTCATGGCCCTTACG |
| *argB*-R | CGGCCTCGATGATATCCATC |
| *argC*-F | CTTCTTCCTGCGGTTCAAGC |
| *argC*-R | GCTGACTTCGCCGAGGTTCT |
| *argD*-F | GCTCATCAAGCGTTTTTCGC |
| *argD*-R | CATTGGCAGGAACGCTTCAC |
| *argF*-F | CGTACTCGCTTCTCCTTCGA |
| *argF*-R | GTGGAAATTGCTGTGTGCGT |
| *argJ*-F | TTGATCAGCTGACCGCTGAG |
| *argJ*-R | CCTGAGTAACGGATGCGTCA |
| 16sRNA-F | AAGAAGCACCGGCTAACTAC |
| 16sRNA-R | CCGGGATTTCACAGACGAC |

The overlapping region with PCR fragment were marked by underline.

TABLE S2 RBS sequences for attenuation of *argF*

| **Name** | **RBS sequence (5’-3’)** |
| --- | --- |
| argF (10 au) | CTATTCCGCTCAACCGAGAATAGCGCCTAAGGGT |
| argF (50 au) | ACTATCTACTCCTATAACCTTAGGTC |
| argF (100 au) | TAGGCGCATAAGGAAAACCGCTCTTTCCGCTTA |
| argF (500 au) | TAGTAATTACACTTTCCACTACTCCTACTA |

TABLE S3 Terminator sequences of attenuation of *argF*

| **Name** | **Terminator sequence (5’-3’)** | **Terminator strength** |
| --- | --- | --- |
| T1(rrnB) | GCTCGAATTCAGCTTGGCTGTTTTGGCGGATGAGAGAAGATTTTCAGCCTGATACAGATTAAATCAGAACGCAGAAGCGGTCTGATAAAACAGAATTTGCCTGGCGGCAGTAGCGCGGTGGTCCCACCTGACCCCATGCCGAACTCAGAAGTGAAACGCCGTAGCGCCGATGGTAGTGTGGGGTCTCCCCATGCGAGAGTAGGGAACTGCCAGGCATCAAATAAAACGAAAGGCTCAGTCGAAAGACTGGGCCTTTCGTTTTATCTGTTGTTTGTCGGTGAACGCTCTCCTGAGTAGGACAAATCCGCCGGGAGCGGATTTGAACGTTGCGAAGCAACGGCCCGGAGGGTGGCGGGCAGGACGCCCGCCATAAACTGCCAGGCATCAAATTAAGCAGAAGGCCATCCTGACGGATGGCCTTTTTGCGTTTCTACAAACTCT | NT |
| T2 | GGAAACACAGAAAAAAGCCCGCACCTGACAGTGCGGGCTTTTTTTTTCGACCAAAGG | 382.13 |
| T3 | CTCGGTACCAAATTCCAGAAAAGAGGCCTCCCGAAAGGGGGGCCTTTTTTCGTTTTGGTCC | 312.50 |
| T4 | CTCGGTACCAAAAAAAAAAAAAAAGACGCTGAAAAGCGTCTTTTTTCGTTTTGGTCC | 309.52 |
| T5 | AAAAAAAAAAAACACCCTAACGGGTGTTTTTTTTTTTTTGGTCTCCC | 281.90 |
| T6 | CTCGGTACCAAATTCCAGAAAAGAGGCCGCGAAAGCGGCCTTTTTTCGTTTTGGTCC | 270.95 |
| T7 | GACGAACAATAAGGCCTCCCAAATCGGGGGGCCTTTTTTATTGATAACAAAA | 243.53 |
| T8 | CTCGGTACCAAATTCCAGAAAAGAGACGCTGAAAAGCGTCTTTTTTCGTTTTGGTCC | 239.91 |
| T9 | CTCGGTACCAAATTCCAGAAAAGAGACGCTTTTAGAGCGTCTTTTTTCGTTTTGGTCC | 216.60 |
| T10 | GACGAACAATAAGGCCTCCCTAACGGGGGGCCTTTTTTATTGATAACAAAA | 177.94 |
| T11 | AACGCATGAGAAAGCCCCCGGAAGATCACCTTCCGGGGGCTTTTTTATTGCGC | 164.60 |
| T12 | CTCGGTACCAAATTCCAGAAAAGACACCCGAAAGGGTGTTTTTTCGTTTTGGTCC | 151.16 |
| T13 | CCAATTATTGAACACCCTAACGGGTGTTTTTTTGTTTCTGGTCTCCC | 128.86 |
| T14 | CCAATTATTGAACACCCAAATCGGGTGTTTTTTTGTTTCTGGTCTCCC | 93.18 |
| T15 | CCAATTATTGAAGCGGCTAACGCCGCTTTTTTTGTTTCTGGTCTCCC | 67.43 |
| T16 | AAGACCCCCGCACCGAAAGGTCCGGGGGTTTTTTTT | 40.39 |
| T17 | GGGCGGTCAGATGATCGCCCTTTTTTTTT | 29.97 |
| T18 | ACGAGCCAATAAAAATACCGGCGTTATGCCGGTATTTTTTTACGAAAGA | 10.94 |
